# Seasonal Influenza and Avian Influenza A(H5N1) Virus Surveillance among Inpatients and Outpatients, East Jakarta, Indonesia, 2011–2014

**DOI:** 10.3201/eid2511.181844

**Published:** 2019-11

**Authors:** Kathryn E. Lafond, Catharina Y. Praptiningsih, Amalya Mangiri, Misriyah Syarif, Romadona Triada, Ester Mulyadi, Chita Septiawati, Vivi Setiawaty, Gina Samaan, Aaron D. Storms, Timothy M. Uyeki, A. Danielle Iuliano

**Affiliations:** University of Tampere, Tampere, Finland (K.E. Lafond);; US Centers for Disease Control and Prevention, Atlanta, Georgia, USA (K.E. Lafond, A.D. Storms, T.M. Uyeki, A.D. Iuliano);; US Centers for Disease Control and Prevention, Jakarta, Indonesia (C.Y. Praptiningsih, A. Mangiri, E. Mulyadi);; Ministry of Health, Jakarta (M. Syarif, R. Triada, C. Septiawati, V. Setiawaty);; Australian National University, Canberra, Capital Territory, Australia (G. Samaan)

**Keywords:** influenza, humans, influenza in birds, seasonal influenza, public health surveillance, viruses, avian influenza A(H5N1) virus, influenza A(H1N1)pdm09, influenza B virus, surveillance, East Jakarta, Indonesia, inpatients, outpatients, influenza-like illness, severe acute respiratory infection, respiratory infections, influenza season, zoonoses, pneumonia, oseltamivir, public health policy, epidemiology

## Abstract

During October 2011–September 2014, we screened respiratory specimens for seasonal and avian influenza A(H5N1) virus infections among outpatients with influenza-like illness and inpatients with severe acute respiratory infection (SARI) in East Jakarta, an Indonesia district with high incidence of H5N1 virus infection among poultry. In total, 31% (1,875/6,008) of influenza-like illness case-patients and 15% (571/3,811) of SARI case-patients tested positive for influenza virus. Influenza A(H1N1)pdm09, influenza A(H3N2), and influenza B virus infections were detected in all 3 years, and the epidemic season extended from November through May. Although 28% (2,810/10,135) of case-patients reported exposure to poultry, only 1 SARI case-patient with an H5N1 virus infection was detected. Therefore, targeted screening among case-patients with high-risk poultry exposures (e.g., a recent visit to a live bird market or close proximity to sick or dead poultry) may be a more efficient routine surveillance strategy for H5N1 virus in these types of settings.

Seasonal influenza contributes substantially to acute respiratory disease in Indonesia and across the world. Influenza virus causes ≈3–5 million cases of severe illness (*1*) and ≈291,000–646,000 respiratory deaths each year globally, most occurring in lower-income countries (*2*). In Indonesia, published data suggest that influenza virus infection has a substantial effect on population health and the healthcare system, causing both inpatient and outpatient respiratory illness (*3*–*5*). Among the more densely populated western and central islands of Indonesia, influenza activity peaks in December and January, correlating with the rainy season. However, limited data from 1 district of Jakarta suggest that a longer peak in influenza activity occurs December–May, with multiple influenza viruses co-circulating (*4*).

In addition to seasonal influenza A and B epidemics, highly pathogenic avian influenza A(H5N1) virus also circulates among poultry in Indonesia (*6*). Jakarta Province is a hub for Indonesia’s commercial poultry trade, and East Jakarta (1 of its 5 districts) is the main entry point for national poultry shipments (*7*). During 2005–2017, Indonesia detected and reported 200 H5N1 infections in humans, of which 168 (84%) were fatal (*8*). Although the number of infections in humans has decreased in Indonesia since 2015, this country still has the second highest number of reported cases (after Egypt) and the highest reported case-fatality proportion among all countries reporting H5N1 virus infections in humans. In East Jakarta, 12 of 13 H5N1 cases reported in humans during 2005–2015 were fatal (*9*).

Although influenza surveillance capacity in Indonesia has increased (*5*,*10*), national policies for influenza vaccination and antiviral use are limited. Influenza vaccination is recommended only for Hajj pilgrims and antivirals only for those with H5N1 virus infection (*11*,*12*). Thus, multiyear data are needed to explore trends in seasonal influenza and avian H5N1 virus infections among humans. Data are particularly needed in regions of Indonesia where highly pathogenic avian influenza A(H5N1) viruses are prevalent among poultry and the risk for poultry-to-human H5N1 virus transmission is elevated. Here, we describe the findings from a 3-year enhanced surveillance platform among inpatients and outpatients of clinics in East Jakarta for both seasonal influenza and avian influenza A(H5N1) viruses.

## Methods

During October 2011–September 2014, we conducted enhanced influenza surveillance in East Jakarta to monitor influenza-like illness (ILI) among case-patients seeking treatment at 4 public primary health centers and severe acute respiratory infection (SARI) among inpatients at 6 hospitals (3 public and 3 private, all equipped with an intensive care unit [ICU]). We selected hospitals on the basis of their location within or bordering the district and the number of persons admitted for respiratory disease. We selected outpatient sites (among 10 subdistrict-level clinics present in East Jakarta) on the basis of proximity to live bird markets. ILI surveillance was conducted Monday–Friday, and surveillance staff used the case definition of recorded temperature >38°C with cough or sore throat to identify ILI case-patients. SARI surveillance was conducted every day in adult and pediatric wards (excluding surgery, obstetric, and gynecology) at all 6 hospitals. Hospital staff used the 2005 World Health Organization (WHO) Integrated Management of Childhood Illness case definition for pneumonia for case-patients <5 years of age (*13*) to identify SARI cases in this age group. To identify SARI in case-patients >5 years of age, staff used the 2011 WHO SARI case definition, which defines SARI as fever (measured temperature >38°C or subjective report of feverishness), disease onset within 7 days, and >1 of the following signs or symptoms: cough, sore throat, or shortness of breath (*14*). We defined elevated respiratory rate as >60 breaths/min for case-patients <2 months of age, >50 breaths/min for case-patients 2–11 months of age, >40 breaths/min for case-patients 1–4 years of age, >35 breaths/min for case-patients 5–7 years of age, >31 breaths/min for case-patients 8–11 years of age, >28 breaths/min for case-patients 12–14 years of age, and >25 breaths/min for case-patients >15 years of age (*15*,*16*). Staff obtained written consent from all enrolled ILI and SARI case-patients; surveillance staff completed case forms for and collected specimens from all enrolled case-patients. We collected data on demographic characteristics, clinical presentation, and exposure to poultry. Poultry exposure questions included questions on the exposures listed in the Indonesia case definition for suspected H5N1 virus infection (i.e., touching healthy, sick, or dead poultry or poultry products; slaughtering or cleaning poultry; or contact with chicken manure), as well as on poultry ownership, visiting live poultry markets, and neighborhood poultry die-offs (*4*). For SARI case-patients, we additionally collected data on risk factors for severe disease (chronic conditions, pregnancy, and smoking status), self-reported influenza vaccine use, and duration and outcome of hospitalization.

Surveillance staff collected nasal and throat swab samples from all enrolled ILI and SARI case-patients and sent them to 2 laboratories in Jakarta for influenza diagnosis (Provincial Health Laboratory of Daerah Khusus Ibukota [DKI] Jakarta and the Infectious Disease Hospital Sulianti Saroso Laboratory). Quality control testing was provided by the National Institute of Health Research and Development, a WHO-designated National Influenza Centre. These laboratories tested samples by real-time reverse transcription PCR (RT-PCR) following the US Centers for Disease Control and Prevention in-house real-time RT-PCR protocol for detection and characterization of influenza viruses. All specimens were tested for influenza A and B viruses, and influenza A virus–positive specimens were further subtyped for influenza A(H1N1)pdm09 and A(H3N2) viruses; specimens that were negative for these subtypes were then tested for H5N1 virus.

All surveillance and laboratory records were entered into a secured web-based electronic reporting system (Surveilans ILI-SARI DKI Jakarta) hosted by the DKI Jakarta Provincial Health Office (Jakarta, Indonesia). All eligible ILI and SARI case-patients were enumerated and reported through this electronic system on a weekly basis. The central project team trained site-level surveillance staff at the beginning of the project and conducted regular monitoring visits throughout the study to ensure thorough case ascertainment and data quality assurance at each site. Complete information on surveillance and data management methods were published previously (*4*).

We analyzed bivariate comparisons using χ^2^ and Fisher exact tests. For time variables (e.g., time from illness onset to seeking care, duration of hospitalization), we determined median values and evaluated differences using the Mann-Whitney U test. We compared influenza virus detection across seasons qualitatively and identified time periods with elevated influenza virus circulation by determining the percentage of influenza virus–positive ILI or SARI episodes among all ILI or SARI episodes each week. We used poultry contact data to determine the percentage of case-patients who met the Indonesia case definition for suspected H5N1 virus infection (i.e., acute lower respiratory tract illness with fever and recent exposure to poultry or a human with a suspected H5N1 virus infection within 7 days of illness onset, based on WHO case definition) (*17*). We defined children as persons <18 years of age. We conducted all statistical analyses using SPSS version 21 (IBM, https://www.ibm.com).

This surveillance project was reviewed and considered to be routine public health surveillance by the Ministry of Health (Jakarta, Indonesia) and US Centers for Disease Control and Prevention (Atlanta, Georgia, USA). Thus, our surveillance was exempted from institutional review board approval.

## Results

During October 2011–September 2014, we enrolled 6,064 ILI case-patients (1% of 476,537 outpatient visits) (Table 1). Most (87%, 5,261/6,064) of these case-patients were <18 years of age. Because of case-patient refusal, we could not collect 56 respiratory specimens (from 1% of those enrolled). Of the 6,008 ILI specimens collected, 1,875 (31%) were positive for influenza virus. Median time from illness onset to outpatient visit among all ILI case-patients was 1 (interquartile range [IQR] 1–2) day (Table 2). Influenza virus–positive ILI case-patients (median age 7 [IQR 3–13] years) were older than influenza virus–negative ILI case-patients (median age 4 [IQR 1–8] years; p<0.001 by χ^2^ test). Among ILI case-patients, influenza virus–negative case-patients (19%) were more likely than influenza virus–positive case-patients (12%; p<0.001) to have an elevated respiratory rate. More than one quarter (29%, 546/1,875) of influenza virus–positive ILI case-patients met the national case definition for suspected H5N1 virus infection.

**Table 1:** Characteristics of ILI and SARI case-patients in study of incidence of seasonal and avian influenza A(H5N1) virus infections, East Jakarta, Indonesia, October 2011–September 2014*

**Characteristic**	**ILI, n = 6,064**	**SARI, n = 4,071**
**Age, y**		
** <5**	2,715 (44.8)	1,414 (35)
** 5–17**	2,546 (42.0)	650 (16)
** 18–49**	705 (11.6)	1,298 (32)
** 50–64**	85 (1.4)	463 (11)
**>65**	13 (0.2)	246 (6)
**Age, y, median (IQR)**	5 (2–9)	17 (2–40)
**Sex**		
** M**	2,930 (48)	2,205 (54)
** F**	3,134 (52)	1,866 (46)
**Specimen collected and tested for influenza**	6,008 (99)	3,811 (94)
**Influenza virus positive **		
** Any**	1,875/6,008 (31)	571/3,811 (15)
** A**	1,057/1,875 (56)	330/571 (58)
** A(H3N2)**	605/1,057 (57)	177/330 (53.6)
** A(H1N1)pdm09**	452/1,057 (43)	152/330 (46.1)
** A(H5N1)**	0	1/330 (0.3)
** B**	827/1,875 (44)	241/571 (42)
**Met Indonesia case definition for suspected avian H5N1 virus infection†**	1,943 (32)	867 (21)

*Values are no. (%), except where indicated otherwise. ILI and SARI were defined as described in the text. ILI, influenza-like illness; IQR, interquartile range; SARI, severe acute respiratory infection.  **†Acute lower respiratory tract illness with fever and recent exposure to poultry (i.e., touching healthy, sick, or dead poultry or poultry products; slaughtering or cleaning poultry; or contact with chicken manure) or a human with a suspected H5N1 virus infection within 7 days of illness onset.**

**Table 2:** Characteristics of ILI case-patients by influenza virus positivity, East Jakarta, Indonesia, October 2011–September 2014*

Characteristic	Influenza virus positive		p value
	Yes, n = 1,875	No, n = 4,133	
Sex			
M	900 (48)	2,007 (49)	0.687
F	975 (52)	2,126 (51)	
Age, y†			
<5	562 (30.0)	2,150 (52.0)	
5–17	915 (48.8)	1,592 (38.5)	
18–49	350 (18.7)	341 (8.3)	
50–64	41 (2.2)	44 (1.1)	
>65	7 (0.4)	6 (0.1)	
Age, y, median (IQR)	7 (3–13)	4 (1–8)	<0.001
Time from illness onset to outpatient visit, d, median (IQR)	1 (1–2)	1 (1–2)	0.526
Elevated respiratory rate‡	229 (12)	796 (19)	<0.001
Met Indonesia case definition for suspected avian influenza A(H5N1) virus infection§	546 (29)	1,387 (34)	<0.001

*Values are no. (%), except where indicated otherwise. ILI was defined as stated in the text. ILI, influenza-like illness; IQR, interquartile range. †Percentages in group do not always add up to 100% because of rounding. ‡Defined as described in the text. §Acute lower respiratory tract illness with fever and recent exposure to poultry (i.e., touching healthy, sick, or dead poultry or poultry products; slaughtering or cleaning poultry; or contact with chicken manure) or a human with a suspected H5N1 virus infection within 7 days of illness onset.

We enrolled 4,071 SARI case-patients (2% of 184,576 hospitalizations) (Table 1). We could not collect 260 (6%) SARI specimens because of case-patient refusal or discharge before specimen collection; thus, we excluded these case-patients. Median time from illness onset to hospital admission was 3 days for children and adults, and median time from illness onset to specimen collection was 5 days (Table 3). Of the 3,811 SARI specimens collected, 571 (15%), including 1 positive for H5N1 virus, were positive for influenza virus (Table 1). Among all influenza virus–positive SARI case-patients, 97 (17%) met the national case definition for suspected H5N1 virus infection (Table 3).

**Table 3:** Characteristics of case-patients with SARI, by influenza virus positivity, East Jakarta, Indonesia, October 2011–September 2014*

Characteristic	Influenza virus positive		p value
	Yes, n = 571	No, n = 3,240	
Sex			0.816
M	310 (54)	1,742 (54)	
F	261 (46)	1,498 (46)	
Age, y†			
<5	164 (29)	1,109 (34)	
5–17	111 (19)	503 (16)	
18–49	186 (33)	1,065 (33)	
50–64	78 (14)	364 (11)	
>65	32 (6)	199 (6)	
Age, y, median (IQR)	21 (4–43)	18 (2–40)	0.019
Time from illness onset to admission, d, median (IQR)	3 (2–4)	3 (2–5)	0.020
Time from illness onset to specimen collection, d, median (IQR)	5 (4–6)	5 (4–7)	0.012
Concurrent medical conditions			
>1	123 (22)	779 (24)	0.195
Asthma	55 (10)	253 (8)	0.294
Tuberculosis	43 (8)	345 (11)	0.045
Diabetes	27 (5)	141 (4)	0.007
Influenza vaccination in past year	2 (0)	36 (1)	0.066
Pregnancy‡	5/93 (5)	22/568 (4)	0.497
Current smoker	75 (13)	466 (14)	0.7
Elevated respiratory rate§	139 (24)	1,061 (33)	<0.001
Chest radiograph–confirmed pneumonia¶	177/481 (37)	1,153/2,799 (41)	0.07
Met Indonesia case definition for suspected influenza A(H5N1) virus infection#	97 (17)	721 (22)	0.005
Sought care before admission**			0.007
Yes	180/276 (65)	1,112/1,514 (73)	
No	96/276 (35)	402/1,514 (27)	
Primary discharge diagnosis available	517 (91)	2,912 (90)	0.625
Pneumonia	132/517 (26)	996/2,912 (34)	<0.001
Typhoid	103/517 (20)	303/2,912 (10)	
Unspecified febrile Illness	57/517 (11)	189/2,912 (6)	
Upper respiratory tract infection	43/517 (8)	235/2,912 (8)	
Tuberculosis	32/517 (6)	311/2,912 (11)	
Discharge form completed	568 (99)	3,211 (99)	0.465
Medication during hospitalization			
Antimicrobial drugs	554/568 (98)	3,093/3,211 (96)	0.14
Oseltamivir	14/568 (2)	22/3,211 (1)	<0.001
Corticosteroid	10/568 (2)	116/3,211 (4)	0.064
Intensive care unit	6/568 (1)	101/3,211 (3)	0.006
Mechanical ventilation	6/568 (1)	76/3,211 (2)	0.049
Length of hospitalization, d, median (IQR)	4 (3–6)	5 (3–8)	<0.001
Outcome			0.075
Recovered	550/568 (97)	3,022/3,211 (94)	
Died	10/568 (2)	103/3,211 (3)	
Other††	8/568 (1)	86/3,211 (3)	

*Values are no. (%), except where indicated. SARI was defined as stated in the text. IQR, interquartile range; SARI, severe acute respiratory infection. †Percentages in group do not always add up to 100% because of rounding. ‡Among female case-patients 15–49 years of age. §Defined as described in the text. ¶Among those with a chest radiograph performed. #Acute lower respiratory tract illness with fever and recent exposure to poultry (i.e., touching healthy, sick, or dead poultry or poultry products; slaughtering or cleaning poultry; or contact with chicken manure) or a human with a suspected H5N1 virus infection within 7 days of illness onset. **Among those for which prehospitalization care-seeking behaviors were known. ††Includes forced discharge and referrals.

The H5N1 virus–positive case-patient was a 33-year-old man (Table 4) with an onset of illness of June 1, 2014. On June 4, he was admitted into a hospital, where he received a diagnosis of pneumonia (chest radiograph data unavailable) and was enrolled into surveillance; his respiratory specimen was collected on June 6. On June 13, he was transferred to a designated referral hospital, where he began antiviral treatment and died on June 14. He was obese but had no other concurrent medical condition; exposure history included visiting a live bird market, where he purchased live poultry within 7 days before hospital admission. This case was not linked to any documented bird outbreaks. No animal or environmental specimen collection occurred because of the length of time that elapsed before linkage to a live poultry market and because of rapid turnover of poultry through these markets.

**Table 4:** Characteristics of SARI case-patients with severe influenza, East Jakarta, Indonesia, October 2011–September 2014*

Case-patient no.	Age, y/sex	Influenza virus detected	Diagnosis	Influenza vaccine in previous year	Underlying conditions	ICU or HCU admission	Outcome
1	50/M	A(H3N2)	Suspected A(H5N1) infection	No	Smoker, diabetes, heart disease	Yes	Recovered
2	33/M	A(H5N1)	Pneumonia	No	Obesity	Unknown	Died
3	83/M	B	Pneumonia	No	Smoker, diabetes, heart disease, asthma	No	Died
4	70/M	A(H3N2)	Pneumonia	No	Smoker	No	Died
5	66/M	A(H1N1)pdm09	Pneumonia	Unknown	Unknown	Yes	Died
6	0.5/F	B	Pneumonia	No	None recorded	Yes	Referred
7	1/M	A(H3N2)	Pneumonia	No	None recorded	Yes	Recovered
8	2/F	A(H1N1)pdm09	Febrile illness	No	None recorded	No	Died
9	64/F	A(H3N2)	Febrile illness	No	Diabetes, heart disease, asthma	No	Died
10	59/M	A(H3N2)	Chronic obstructive pulmonary disease	No	Smoker, kidney disease, asthma	No	Died
11	62/M	A(H3N2)	Congestive heart failure exacerbation	No	Smoker, heart disease, chronic lung disease	No	Died
12	39/M	A(H1N1)pdm09	Suspected A(H5N1) infection	No	Smoker	Yes	Died
13	63/F	A(H1N1)pdm09	Tuberculosis	No	Tuberculosis, asthma	No	Died
14	1/M	A(H1N1)pdm09	Anemia, malnutrition	No	None recorded	Yes	Recovered

*SARI was defined as stated in the text. SARI case-patients with severe influenza were those who were admitted into the HCU or ICU, died, or both as a result of influenza virus infection. HCU, high care unit; ICU, intensive care unit; SARI, severe acute respiratory infection.

SARI case-patients were 0–90 years of age. A higher proportion of SARI cases among children 5–17 years of age and adults 50–64 years of age were influenza virus–positive compared with other age groups (p = 0.016 by χ^2^ test) (Table 3). Adults >65 years of age comprised 6% of all influenza virus–positive and influenza virus–negative SARI cases. Concurrent medical conditions, such as tuberculosis (10%, 388/3,811), asthma (8%, 308/3,811), and diabetes (4%, 168/3,811), were reported among 24% (902/3,811) of SARI case-patients; of these conditions, only diabetes was more commonly associated with influenza virus infection (p = 0.007). Data on healthcare seeking before admission were available for about half (46%, 1,790/3,811) of SARI case-patients. Influenza virus–negative SARI case-patients (73%, 1,112/1,514) were more likely than influenza virus–positive case-patients (65%, 180/276) to have sought care before hospitalization (p = 0.007). Overall, 38 (1%) SARI case-patients reported receiving the influenza vaccine in the previous 12 months, of whom 36 (95%) had a negative influenza virus test finding.

Chest radiograph–confirmed pneumonia was documented for 35% (1,330/3,811) of SARI case-patients (Table 3). Of case-patients with known primary discharge diagnoses, pneumonia (33%, 1,128/3,429) was the most common diagnosis, followed by typhoid (12%, 406/3,429). Almost all (96%, 3,647/3,811) SARI case-patients received antibiotics during hospitalization. Only 2% (14/568) of influenza virus–positive SARI case-patients and 1% (22/3,211) of influenza virus–negative case-patients with data on medication use were treated with oseltamivir. Median duration of hospital stay was slightly longer for adults (5 days) than children (4 days; p<0.001) and influenza virus–negative (5 days) than influenza virus–positive (4 days; p<0.001) case-patients. Overall, 3% (107/3,779) of case-patients with a completed discharge form had been admitted to the ICU; ICU admission was less common among influenza virus–positive case-patients (1%, 6/568) than influenza virus–negative case-patients (3%, 101/3,211; p = 0.006). Of the 6 case-patients with influenza who were admitted to an ICU, 3 were children and 3 were adults (Table 4). One of these adult case-patients had concurrent medical conditions, and 2 were smokers. At the time of discharge, 95% (3,572/3,779) of SARI case-patients had recovered (Table 3). In total, 113 SARI case-patients died; 10 were positive for influenza virus. Of those 10 deaths, 9 were associated with seasonal influenza viruses and 1 with H5N1 virus. Two SARI case-patients with severe influenza had a discharge diagnosis of suspected H5N1; both had seasonal influenza virus infection.

Collectively among ILI and SARI case-patients, influenza A(H1N1)pdm09, influenza A(H3N2), and influenza B viruses were detected in all 3 years (Figure). The percentage of ILI case-patients who were influenza virus positive during year 1 (35%, 1,131/3,278) was slightly higher than the percentages positive during years 2 (27%, 427/1,562) and 3 (27%, 317/1,168). The percentage of SARI case-patients who were influenza virus positive was consistent across all 3 years: 15% (276/1,787) in year 1, 14% (153/1,122) in year 2, and 16% (142/902) in year 3. Each year, the percentage of samples positive for influenza virus per week started increasing in November, peaked in January or February, and declined in March but persisted through May. Influenza B virus infections were typically identified later in each season. Each year, the rate of influenza virus–positive samples per week peaked around 72%–85% for ILI and 38%–50% for SARI.

**Figure:**
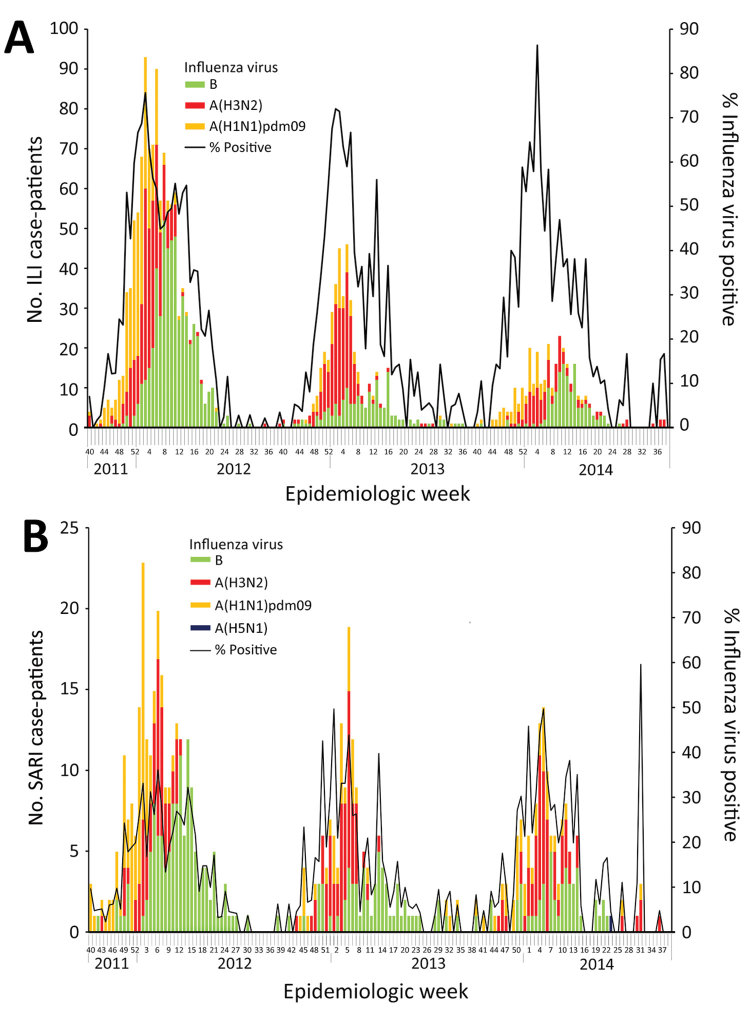
Seasonal and avian influenza A(H5N1) virus–positive ILI (A) and SARI (B) case-patients by clinical presentation and epidemiologic week, East Jakarta, Indonesia, October 2011–September 2014. ILI and SARI were defined as stated in the text. ILI, influenza-like illness; SARI, severe acute respiratory infection.

## Discussion

During this 3-year period of enhanced surveillance, influenza viruses were commonly detected among ILI and SARI case-patients in East Jakarta, representing 31% of ILI visits and 15% of SARI hospitalizations, many of which were chest radiograph–confirmed pneumonia. Although many persons met the Indonesia case definition for suspected avian H5N1 virus infection because of their interaction with poultry, only 1 case of H5N1 virus infection was identified. Within 7 days before illness onset, this H5N1 case-patient had visited a live bird market, a type of poultry exposure reported among other persons with H5N1 virus infection in Indonesia; this type of exposure was reported among only 4% of SARI case-patients in this surveillance.

One of the primary objectives of this project was to systematically test both inpatients and outpatients for H5N1 virus infection. Although the case-fatality proportion reported for humans with H5N1 virus infection in Indonesia is high, underdetection in less severely ill patients was still a possibility. Given that fatal outcomes are relatively rare, the ubiquity of exposure to poultry in parts of this country prohibits rigorous screening of all respiratory patients for H5N1 virus infection by sophisticated laboratory diagnostic methods such as RT-PCR. Our detection of only 1 case-patient with H5N1 virus infection in this high-risk district with endemic H5N1 virus circulation among poultry provides evidence to suggest that the likelihood of underascertainment in high-risk areas of Indonesia is low. Routine influenza surveillance in Indonesia involves inpatient and outpatient facilities, identification of SARI and ILI cases, and RT-PCR diagnostic testing; however, this surveillance is not specifically targeted to areas with endemic H5N1 virus circulation, and the volume of samples tested is much lower than the volume tested in this project.

Contrary to the infrequent detection of avian H5N1 virus, seasonal influenza A and B viruses were major contributors to medically treated respiratory illnesses in this population. Influenza A(H3N2), influenza A(H1N1)pdm09, and influenza B viruses were all identified, and no single virus type or influenza A virus subtype predominated in any year. In this region, influenza A and B viruses were most frequently detected during the beginning of each calendar year during the rainy season. Months of peak seasonal influenza virus circulation were consistent across all 3 years and covered at least half of each year. These long seasons comprised consecutive peaks of influenza A and B viruses, similar to the pattern seen in US surveillance systems (*18*). The correspondence of influenza virus circulation with the rainy season is also consistent with evidence from other tropical countries of southern and southeastern Asia, including India and Thailand (*19*), as well as with previous outpatient influenza surveillance data from other parts of Indonesia (*3*).

In the study population, ILI case-patients were typically young children, and SARI case-patients included both children and adults. This observation is consistent with the age distribution reported by influenza surveillance platforms in other countries of Asia, such as the Philippines and Mongolia (*20*,*21*). Although older adults are more frequently identified in inpatient surveillance platforms in high-income settings (*22*), adults >65 years of age comprised only 6% of SARI case-patients in our platform. Several factors should be considered when interpreting this finding. First, this finding is consistent with the relatively young age structure of the Jakarta population, where only 5% of persons are >60 years of age (*23*). In addition, we conducted routine monitoring and validation activities to ensure enrollment of all eligible SARI case-patients, so the low number of case-patients enrolled in this age group is unlikely to be attributable to underenrollment. Instead, this trend might reflect differences in clinical presentation of older adults (who might not always meet the SARI case definition), decreased healthcare seeking by older adults, or a high threshold for admission by physicians. In this community, we previously conducted a survey on healthcare-seeking practices and found reported hospitalizations to be higher in older adults than younger adults and children, although the older adult population comprised a small proportion of the surveyed population (*24*).

In addition to age distribution differences between inpatients and outpatients, influenza virus detection status also differed by age. The youngest children with ILI and SARI were less likely to be influenza virus positive; influenza virus detection was more frequent among older children and adults. This trend is consistent with findings from a global meta-analysis on pediatric influenza hospitalizations (*25*) and is likely driven by the high prevalence of respiratory syncytial virus among children <1 year of age; this virus causes a large proportion of hospitalizations among children <5 years of age. Although we did not test respiratory specimens for respiratory syncytial virus, this pathogen has been shown to differentially infect young children who are also at high risk for SARI.

Less than one quarter of influenza virus–positive SARI case-patients reported underlying conditions, such as asthma, tuberculosis, or diabetes, and a small number of SARI case-patients were pregnant women. These findings might be a reflection of care-seeking patterns, particularly of pregnant women, who might prefer to receive care in private maternity hospitals. Our observation of a low percentage of pregnant women with influenza is in contrast with other published findings, such as those from routine SARI surveillance in South Africa, where pregnancy was identified as a risk factor for influenza-associated hospitalization (*26*). Concurrent medical conditions were also more prevalent among influenza virus–positive SARI case-patients in South Africa (35%), although surveillance in other locations, such as Damanhour, Egypt (*27*), revealed lower prevalences of concurrent conditions (19%) than that found in our study (22%). These differences might reflect baseline population variability in prevalence of diseases or variability in the types of persons seeking care at the facilities participating in surveillance.

Although use of antibiotics was common among patients in this study, only 2% of influenza virus–positive and 1% of influenza virus–negative SARI case-patients received antiviral treatment with oseltamivir. Oseltamivir is typically prescribed by physicians who suspect their patient has an H5N1 virus infection, per the Indonesia national policy for antiviral use (*12*). However, treating hospitalized influenza patients with oseltamivir is clinically beneficial regardless of whether the infection is seasonal or pandemic influenza virus, and recommendations outside of Indonesia include initiation of antiviral treatment as soon as possible for hospitalized patients with seasonal influenza (*28*,*29*). Likewise, the report of influenza vaccination among case-patients was low, probably because of the lack of a national influenza vaccination program in Indonesia, aside from the recommendation for vaccination for Hajj travelers.

The surveillance platform in this study, involving an urban district within the capital province of Jakarta, was specifically selected for its high prevalence of H5N1 viruses in poultry (*30*); therefore, our findings cannot be generalized to all of Indonesia. Although routine surveillance in other districts might potentially detect even lower percentages of patients with H5N1 virus infection, likelihood of detection through a surveillance system varies by location and over time, and the true risk for infection cannot be known. Confirmed H5N1 virus infections in humans decreased in Indonesia in the 2010s; infections peaked in 2006 with 55 cases, and only 3 cases were identified during 2015–2018. This decrease in H5N1 virus infections in humans has also been observed in other countries where the virus is endemic in poultry. In addition, only a fraction of patients with seasonal influenza or avian H5N1 virus infection will seek care, and those who do seek care might not meet the typical surveillance case definition or might no longer be shedding detectable influenza virus in the upper respiratory tract by the time they provide a respiratory specimen. Avian H5N1 viruses, in particular, preferentially bind receptors in the lower respiratory tract and can be missed in upper respiratory tract specimen collection. Last, our results are reliant on self-report of risk factors, such as poultry exposure and chronic medical conditions, meaning we could be underestimating the frequency of these risk factors, although recall is unlikely to differ between those with and without influenza virus infection. Despite these limitations, we believe our findings provide valuable insight into patterns of seasonal influenza virus circulation and severe respiratory disease in this urban setting in Indonesia.

In conclusion, seasonal influenza was found to be a key contributor to outpatient and inpatient respiratory disease in this urban setting of Indonesia, where avian H5N1 viruses are frequently detected among poultry. In contrast, avian H5N1 virus infection was only detected in 1 SARI case-patient, despite rigorous and systematic screening of both inpatients with SARI and outpatients with ILI. In settings like East Jakarta, where poultry exposure is common, our findings support restricting RT-PCR testing for H5N1 viruses to sick patients with unsubtypeable influenza A virus infection and those with high-risk poultry exposures, such as a recent visit to a live bird market or close proximity to sick or dead poultry. Knowledge about the prevalence of seasonal influenza and timing of the local influenza season could also be leveraged by policy makers, public health officials, and healthcare providers to improve risk communication and develop appropriate prevention and control measures, such as early empiric antiviral use and optimally timed influenza vaccination activities.
